# Circulating Long Non-Coding RNAs as Novel Potential Biomarkers for Osteogenic Sarcoma

**DOI:** 10.3390/cancers13164214

**Published:** 2021-08-21

**Authors:** Sutpirat Moonmuang, Parunya Chaiyawat, Salinee Jantrapirom, Dumnoensun Pruksakorn, Luca Lo Piccolo

**Affiliations:** 1 Center of Multidisciplinary Technology for Advanced Medicine (CMUTEAM), Faculty of Medicine, Chiang Mai University, Chiang Mai 50200, Thailand; sutpirat_m@cmu.ac.th (S.M.); parunya.chaiyawat@cmu.ac.th (P.C.); 2 Musculoskeletal Science and Translational Research Center (MSTR), Faculty of Medicine, Chiang Mai University, Muang, Chiang Mai 50200, Thailand; 3 Drosophila Center for Human Diseases and Drug Discovery (DHD), Faculty of Medicine, Chiang Mai University, Chiang Mai 50200, Thailand; salinee.jan@cmu.ac.th; 4 Department of Pharmacology, Faculty of Medicine, Chiang Mai University, Chiang Mai 50200, Thailand; 5 Department of Orthopedics, Faculty of Medicine, Chiang Mai University, 110 Intawaroros, Sriphoom, Muang, Chiang Mai 50200, Thailand

**Keywords:** liquid biopsy, circulating long non-coding RNA, osteosarcoma, biomarkers

## Abstract

**Simple Summary:**

Long non-coding RNAs (lncRNAs) can be detected in a liquid biopsy. We herein discussed the origin, methods of detection, measurement and potential functions of lncRNAs in blood. Furthermore, we used a systematic literature search to identify thirteen circulating lncRNAs whose expression was associated with bone tumor and we examined their impacts on clinical decision-making in the management of osteosarcoma.

**Abstract:**

Circulating cell-free nucleic acids recently became attractive targets to develop non-invasive diagnostic tools for cancer detection. Along with DNA and mRNAs, transcripts lacking coding potential (non-coding RNAs, ncRNAs) directly involved in the process of tumor pathogenesis have been recently detected in liquid biopsies. Interestingly, circulating ncRNAs exhibit specific expression patterns associated with cancer and suggest their role as novel biomarkers. However, the potential of circulating long ncRNAs (c-lncRNAs) to be markers in osteosarcoma (OS) is still elusive. In this study we performed a systematic review to identify thirteen c-lncRNAs whose altered expression in blood associate with OS. We herein discuss the potential impact that these c-lncRNAs may have on clinical decision-making in the management of OS. Overall, we aimed to provide novel insights that can contribute to the development of future precision medicine in oncology.

## 1. Introduction

Osteosarcoma (OS) is a highly aggressive malignant bone tumor, frequently occurring in children and adolescents with an annual incidence of over three per million worldwide [1,2,3]. OS represents different pathological entities based on clinical, radiological, and histopathological features. For instance, based on histopathological features, osteosarcoma can be classified into distinct subtypes with the osteoblastic, chondroblastic, and fibroblastic OS, respectively, being the most common [4]. 

Nowadays, various clinical practices for OS have been notably implemented, including chemotherapy, radiotherapy, surgery, and targeted therapy; yet, the prognosis for OS still remains poor [5,6]. In fact, approximately 20% of patients showed clinical metastasis at presentation, with a 5-year survival rate less than 30% [7]. For this reason, OS strongly demands reliable, non-invasive, and clinically useful biomarkers. 

In contrast to conventional biopsy, the liquid biopsy of tumor components in blood represents a simple and rapid test, easily performed, and requiring a small amount of sample (usually 10–15 mL of blood). Presently, however, the usefulness of alkaline phosphatase (ALP) and lactate dehydrogenase (LDH) as laboratory markers for OS is still considered controversial [8,9]. Likewise, studies have shown that programmed cell death 1 ligand-1 (PD-L1) and bone resorption markers, such as b-isomerized C-terminal telopeptides (b-CTx) and total procollagen type 1 amino-terminal propetide (tP1NP), still require more investigation before being able to conclude their potential value as biomarkers for OS [6,10,11,12]. 

Recently, circulating biomarkers, such as circulating tumor cells (CTCs) and different forms of circulating-free and extracellular vesicle/platelet-encapsulated non-coding RNA, including microRNA (miRNA) and long non-coding RNA (lncRNA), have emerged as novel promising diagnostic, prognostic, or predictive biomarkers in the clinical management of patients with OS [13,14,15,16,17,18].

Although CTCs may provide tumor-specific genomic, transcriptomic, and proteomic information, their analysis requires a large volume of fresh blood and it is laborious and expensive. On the other hands, the use of circulating ncRNAs, in spite of some obvious limitations, is more accessible, cheaper, and has shown potential as a precision medicine biomarker [19]. Early studies on circulating RNAs focused on the relevance of miRNAs. However, the current search for novel OS biomarkers has possibly shifted to lncRNAs due to their relative abundance and higher stability with respect to miRNAs [14]. 

Interestingly, a number of circulating lncRNAs, whose expression in liquid biopsy correlate with that of cancer tissues, have emerged as novel diagnostic or prognostic markers for several types of cancer [20,21,22,23]. However, the role of circulating lncRNAs as biomarker for OS is still elusive. In this study, we performed a systematic review to identify, evaluate, and summarize the findings of all relevant studies about circulating lncRNAs that associate with OS progression. We aimed to investigate whether circulating lncRNAs can be employed as novel biomarkers in OS from early cancer detection to therapy selection and cancer patient monitoring during the course of disease.

## 2. Long Non-Coding RNA Structures and Functions

LncRNAs are conventionally classified as transcripts longer than 200 nt with no or low coding potential [24,25,26]. Similar to protein-coding transcripts, the transcription of lncRNAs is dependent on histone-modification-mediated regulation, and lncRNA’s transcripts are processed by the canonical spliceosome machinery. Overall, lncRNA genes show fewer exons than mRNAs, and appear to be under a weaker selective pressure during evolution. Moreover, some lncRNAs are expressed at levels lower than those of mRNAs and in a more tissue- and cell-specific manner, while others are known to be fairly abundant and are expressed in diverse cell types, such as the “house-keeping” genes [27]. 

Of tens of thousands of metazoan lncRNAs discovered from cDNA libraries and RNAseq data by high throughput transcriptome projects, only a handful of lncRNAs have been functionally characterized. The investigations on this small cohort of lncRNAs have demonstrated that these noncoding transcripts can serve as scaffolds or guides to regulate protein–protein or protein–DNA interactions [28,29,30,31] or can modulate post-translational modification of nonhistone proteins [32]. Moreover, lncRNAs are capable of controlling microRNAs (miRNAs) [33,34,35], and function as enhancers to influence gene transcription, when transcribed from the enhancer regions (enhancer RNA) [36,37,38] or their neighboring loci (noncoding RNA activator) [39,40].

Several lines of evidence have shown that lncRNAs are capable of influencing different cellular functions that are critical to tumorigenesis, such as cell proliferation, differentiation, migration, immune response, and apoptosis [41,42,43,44,45,46,47]. Furthermore, lncRNAs have been found to act as tumor suppressors or oncogenes [48,49,50,51], and, of note, a number of lncRNAs have been reported to be significantly deregulated in tumors [52,53,54,55].

## 3. Origin of Circulating lncRNAs

**Figure 1:**
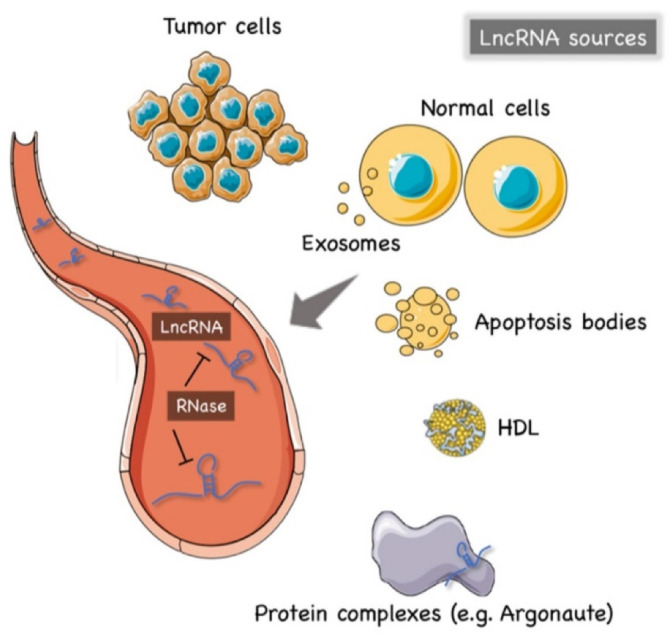
The origin of circulating lncRNAs. Two major sources of circulating lncRNAs have been postulated so far. LncRNAs can be encapsulated in extracellular vesicles (EV), predominantly exosomes. On the other hand, lncRNAs can also be released from live cells in an EV-independent fashion, thus, similar to circulating miRNAs, circulating lncRNAs might be detected in complexes with protein or high-density lipoproteins (HDL). The latter mechanism is likely to offer = circulating lncRNAs less protection against ribonucleases that are normally present in the extracellular space and body fluids. Created by Servier Medical Art (http://smart.servier.com/ (accessed on 15 May 2021)), licensed under Creative Commons Attribution 3.0 Unported License (https://creativecommons.org/licenses/by/3.0/ (accessed on 15 May 2021)).

The precise mechanism of lncRNAs release into the extracellular environment is not completely understood. Hypotheses have arisen that tumor cells, cancer-adjacent normal cells, immune cells, and other blood cells may all release lncRNAs [56,57], as shown in Figure 1. A few studies reported that lncRNAs can be encapsulated into membrane vesicles, such as exosomes or microvesicles (EV), prior to being secreted extracellularly. In such a conformation, the circulating lncRNAs have shown a higher degree stability, probably due to EVs offering protection against the nuclease-mediated degradation that may occur in the extracellular space and in body fluids [20,58,59] (Figure 1). On the other hand, other studies have suggested that the secretion pathway of lncRNAs may also occur in a similar manner to that for miRNAs. As such, lncRNAs might also be released into body fluids in an EV-independent fashion as complexes with high-density lipoproteins (HDLs) or protein Argonaute 2 (AGO2) [60] (Figure 1).

The hypothesis of an EV-independent mechanism for lncRNAs secretion might seem less likely given the high abundance of ribonucleases in serum, plasma, and other bodily fluids that can dramatically affect the stability of lncRNAs in the extracellular environment. However, one can speculate that circulating lncRNAs can be capable of resisting the RNase activity through modifications such as methylation, adenylation, and uridylation [61] or via the formation of higher order structures [62]. 

## 4. Detection Methods of Circulating lncRNAs

Difference sources of liquid biopsy (i.e., whole blood, plasma, serum, urine, and gastric juice) can be used to quantify circulating lncRNAs. However, due to the possibility of blood cell RNA contamination, whole blood is the less recommended option so far [63]. In addition, EDTA-anticoagulant collecting tubes have been suggested to be more suitable for the analysis of circulating lncRNAs [57]. Of note, some studies have found that lncRNAs remained stable in plasma even under multiple cycles of freeze–thaw, incubation at 45 °C, or storage at room temperature for as long as 24 h [56].

Overall, the methods to extract circulating lncRNAs can be divided into two major groups: guanidine/phenol/chloroform-based and column-based protocols. The column-based method is currently considered more reliable, since organic and phenolic contaminants in TRIzol-based methods might invalidate results [64]. 

Regarding the measuring and normalization methods, some studies have suggested that the use of an equal volume of input RNA sample may be more accurate than an equal amounts of RNA measured using a NanoDrop spectrophotometer since many diseases, including cancer, may indeed release an higher degree of RNAs into body fluids than healthy control groups, leading to a significantly higher level of circulating RNA in cancer patients that causes misleading results [64].

To date, quantitative real-time PCR (qRT-PCR) is still considered the gold standard for quantitative expression analysis of lncRNAs, including circulating lncRNAs [65]. Microarrays and whole transcriptome analysis (RNA-seq) still have limited uses in this field. In fact, the high throughput potential of microarrays relies on a reference database of targets, which in the case of circulating lncRNAs, is still very limited [21]; the RNA-seq requires huge amounts of starting RNA samples. Additionally, RNA-seq is currently expensive and needs special equipment and/or expert bioinformaticians [64], whereas, the targeted-approach of qRT-PCR is still more accessible, and saves money and time. Accordingly, qRT-PCR can be divided into relative and absolute analyses. In relative quantification methods, the choice of endogenous controls is critical to properly normalize the expression levels. In this regard, it must be noted that no systematic evaluation of reference genes for serum lncRNA has yet been reported, posing some limitations for the relative qRT-PCR method in the analysis of lncRNAs from a liquid biopsy.

## 5. A Systematic Literature Search Identifies Thirteen Circulating lncRNAs with High Diagnostic Sensitivity in OS

To identify all the circulating lncRNAs whose expression has been reportedly associated with OS, either onset or progression, we ruled out a study in accordance with the preferred guidelines for reporting items for systematic reviews and meta-analyses (PRISMA) [66]. The study protocol of this systematic review was prospectively registered at the international prospective register of systematic review, PROSPERO (CRD42021250424). Briefly, a computerized literature search was performed in PubMed, Embase, and Scopus (last search: May 2021) using the terms “long non coding RNA” or “long untranslated RNA” or “lncRNA” and osteosarcoma and “liquid biopsy” or “serum” or “blood” or “plasma” AND “diagnostic” or “prognosis” or “prognostic” or “survival” or “metastasis”. We further applied inclusion and exclusion criteria as described in the registered protocol. 

**Figure 2:**
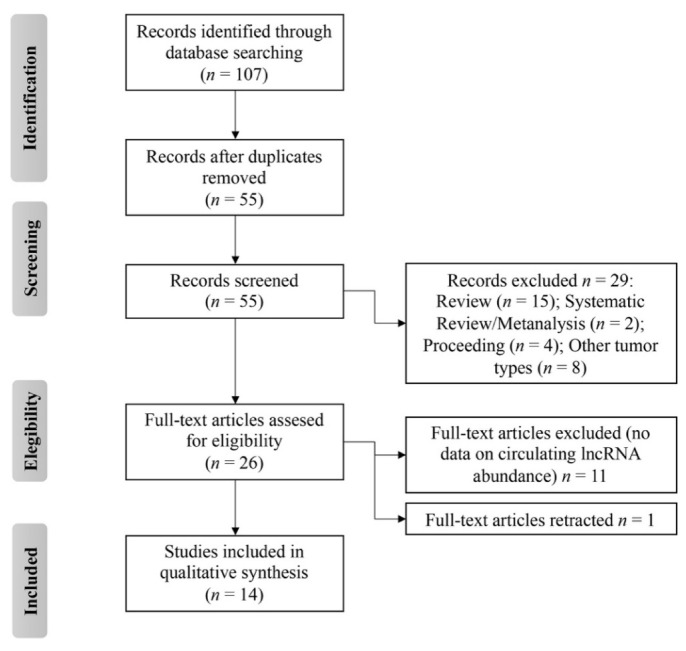
Flow diagram of preferred reporting items for systematic reviews and meta-analyses (PRISMA) of this systematic review.

As a result, a total of 14 studies were identified (Figure 2) to describe the transcript abundance of 13 circulating lncRNAs in OS patients with respect to healthy controls [67,68,69,70,71,72,73,74,75,76,77,78,79,80]. Information pertaining to the methods employed for lncRNA extraction, measurement, and normalization along with their diagnostic and prognostic values were extracted and are listed in Table 1.

To investigate the diagnostic and/or prognostic values of circulating lncRNAs, all the studies enrolled a number of patients and controls in a 1:1 ratio (62 ± 28 participants, mean ± SD), with a total of 873 OS patients who underwent liquid biopsy analysis for the detection of specific circulating lncRNAs, from 2015 to 2021. Altogether, at the time of OS diagnosis, an increase in *ATB*, *EPEL*, *FAL1*, *FGD5-AS1*, *HNF1A-AS1*, *LINC01278*, *LINC01354*, *MALAT1*, *TUG1*, *UCA1* and a decline in *HAND2-AS1* and *NEF* lncRNA expression levels were recorded in patient blood specimens with respect to controls.

Briefly, all studies except that of Jiang (2020) [70] reported the diagnostic value of circulating lncRNAs, while only three studies investigated whether the expression level of circulating lncRNAs change along with disease status of OS patients (pre-operative and post-operative) (Cai 2017 [73], Ma 2015 [80], Wang 2017 [72]). Correlation between the abundance of circulating lncRNAs and survival rate was also measured in five studies (Chen 2018 [68], Huo 2017 [78], Sheng 2019 [74], Song 2020 [79], and Zhang 2021 [69] (Table 1)). Of note, a positive correlation between the transcript levels measured in the bloodstream and OS tissue was found.

Overall, we noticed that serum, either fresh or frozen, was the most common liquid biopsy to study circulating lncRNAs. Primarily, total RNA was extracted using TRIzol reagent and was analyzed using quantitative real-time polymerase chain reaction (RT-qPCR). The abundance of circulating lncRNAs was normalized with respect to either *GAPDH* (six studies) or *β-actin* (five studies) housekeeping transcript levels (Table 1). 

Different statistical methodologies were applied for assessing the relationship between the clinicopathological parameters of patients and the abundance of certain circulating lncRNAs. Overall, receiver–operating characteristic (ROC) curves were used to evaluate the performance of each lncRNA to discriminate OS patients from controls (reported as area under the curve (AUC), in Table 1). All 13 lncRNAs identified in liquid biopsy showed high diagnostic potential with the long intergenic non-coding RNA *LINC01278* being the best performer (AUC = 0.945; 95% CI = 0.908–0.982, *p* value <0.001) (Zhang 2021 [69]). Furthermore, three studies reported the diagnostic power of circulating *HNF1A-AS1* (Cai 2017 [73]), *FAL1* (Wang 2017 [72]) and *MALAT1* (Huo 2017 [77]) lncRNAs to be more effective than alkaline phosphatase (ALP) in distinguishing osteosarcoma from healthy individuals. Notably, Huo (2017 [77]) showed that combined detection of MALAT1 and alkaline phosphatase (ALP) significantly increased diagnostic sensitivity (Table 1). 

The expression levels of HNF1A-AS1, TUG1, and FAL1 were the only ones to be monitored during the course of disease so far. Notably, both studies showed an augmentation of lncRNA expression to be associated with relapse.

Finally, an increase of EPEL (Chen 2019 [76]), MALAT1 (Huo 2019 [77]), TUG1 (Sheng 2019 [74]), and FGD5-AS1 (Song 2020 [79]) or a decline in LINC01278 (Zhang 2021 [69]) levels, respectively were found to be associated with a poor prognosis in OS.

## 6. Circulating lncRNAs Associating with OS Show High Degree of Heterogeneity

Genome browsers for research in comparative genomics, evolution, sequence variation, and transcriptional regulation were used to further identify additional information with respect to the thirteen retrieved lncRNAs. Results were extracted and are listed in Table 2. 

Here, we found that the majority of the circulating lncRNAs that have been studied for their potential as biomarkers for OS, so far, are very large (above 2000 nucleotides (nt)) with a complex transcriptional organization that produces several different splicing variants (SVs). The longest lncRNA identified was *MALAT1*, of which the primary sequence is 8779 nt and produces three SVs, while *HAND2-AS1* shows the most varied transcriptional regulation, being the gene with the largest number of exons in the group, producing eleven SVs (Table 2). In contrast, *FAL1* is the shortest lncRNA (566 nt) consisting of only one SV. Notably, whether only specific SVs are sorted in the secretory pathway to reach the bloodstream is still unknown. 

The sub-cellular localization of retrieved lncRNAs was also studied and an interesting picture emerged: not only were cytoplasmic-located lncRNAs found in the bloodstream (*ATB*, *HNF1A-AS1*, *LINC01278*, *LINC01354*, *LINK-A*, *UCA1*), but also the nuclear-limited *EPEL*, *FAL1*, *FGD5-AS1*, *MALAT1* and *NEF*. Additionally, the lncRNAs, *HAND2-AS1* and *TUG1*, were detected in both cellular compartments, as shown by fluorescence in situ hybridization (FISH) [81,82,83,84].

Although the molecular mechanism to produce circulating lncRNAs is still poorly characterized and their biological significance remains elusive, the secretion of circulating cytoplasmic lncRNAs (cc-lncRNAs) is thought to be similar to that underlying miRNA export, which is based on active secretion mediated by membrane-bound vesicles or through a vesicle-free RNA-binding protein dependent pathway [60,85,86]. Instead, circulating nuclear lncRNAs (cn-lncRNAs) might primarily originate from the passive leakage of dead cells. In this scenario, *ATB*, *HNF1A-AS1*, *LINC01278*, *LINC01354*, *LINK-A* and *UCA1* could play active roles in cell-to-cell communication that might be relevant to disease progression and be worth future investigation.

The presence of a poly-adenylation (A) tail has been documented only for six of the above-mentioned lncRNAs. In fact, evidence shows that *EPEL*, *HAND2-AS1*, *TUG1*, *UCA1* carry sequence motifs recognized by the RNA cleavage complex, while *ATB* and *MALAT1* do not present any typical signal [82,87,88,89].

Finally, three major classes of lncRNAs have been identified in OS patient blood, including four antisense (EPEL, FGD5-AS1, HAND2-AS1, HNF1A-AS1), three competitive endogenous RNAs (ceRNAs) (ATB, LINC01278, LINK-A) and three scaffolds (FAL1, LINC01354, MALAT1). Furthermore, studies have shown that NEF and TUG1 can serve both as a scaffold and ceRNAs [90,91,92,93,94] (Table 2). However, whether similar modes of action are retained by circulating lncRNAs upon contact with any recipient cells is yet unknown. In fact, studies have primarily focused on the potential role of circulating lncRNAs as biomarkers of human diseases, irrespective of their contribution to the pathology. Investigations on this matter would extend our understanding of the biological significance of circulating lncRNAs in OS and other human diseases. 

## 7. The lncRNAs-miRNAs Crosstalk Is Critical for the Biological Activities of Osteosarcoma Cells

Regardless the lncRNA’s presence in the bloodstream, which is relevant to address their potential as markers of OS, evidence of how the thirteen retrieved lncRNAs are involved in OS were also researched and is listed in Table 2. This information might be helpful to further address hypotheses regarding the roles of circulating lncRNAs in the oncogenesis of bone. Despite all the lncRNAs identified herein being previously associated with several other cancer types (Table 2), the mode of how they behave in OS to drive or contribute to pathogenesis remains poorly characterized.

When the thirteen lncRNAs were pooled together and qualitatively analyzed, a lncRNA–miRNAs regulatory axis emerged as the most prominent network associating with the OS pathogenic mechanism, with *ATB*, *FGD5*, *HNF1A-AS1*, *LINC01278*, *MALAT1*, *NEF*, *TUG1* and *UCA1* lncRNAs being reported for their ability to sponge miRNAs that affect a plethora of cellular targets critical in malignancy (Table 2). Interestingly, according to the available data, the regulation of gene expression by a competitive endogenous RNA (ceRNA) mechanism is indeed emerging as a leading lncRNA function in OS, as well as in a remarkable number of other types of cancer (Table 2) (reviewed in [140,141]). For instance, *MALAT1* regulates osteosarcoma progression and facilitates lung metastasis by targeting several miRNA families [142,143,144], promotes thyroid cancer progression by regulating *miR-204* [145], and leads to chemoresistance in hepatocellular carcinoma by sponging *miR-140-5p* [146]. A total of 17 miRNAs have been identified to be downregulated by *MALAT1* in a fashion that is critical to drive OS, such as *miR-202* [144], *miR-206* [142], and *miR-26a-5p* [147] to mention a few.

Moreover, the ability of *EPEL*, *FAL1*, *HAND2-AS1*, *HNF1A-AS1*, *LINC01354*, *LINK-A*, *MALAT1* and *UCA1* lncRNAs to modulate the abundance of transcriptional factors, structural proteins, or to affect key cellular pathways, such as Wnt/β-catenin and PTEN/AKT, have been also described [68,70,71,72,78,115,148,149]. In this regard, Wnt signaling emerged as most regulated by the retrieved circulating lncRNAs. However, whether the regulation of such targets is similarly driven by lncRNAs–miRNAs crosstalk or involves a more complex network is still unknown. Nonetheless, due to the large flexibility to form a variety of different complexes, either with proteins or nucleic acids, it is not surprising that a lncRNA can play different roles at once. For instance, in vitro and in vivo experiments highlighted *MALAT1* and *UCA1*’s capabilities to either regulate miRNA expression or scaffold transcriptional regulators in bone oncogenesis [78,150,151,152].

## 8. Concluding Remarks

The role of lncRNAs in OS tumor development has only recently been investigated, yet several studies have shown that the deregulation of a number of lncRNAs influence the occurrence and progression of osteosarcoma, as reviewed in [153].

Many of these lncRNAs proved to have a detectable expression levels in either serum or plasma samples, making them promising biomarker candidates for non-invasive diagnostics. However, the clinical application of the so-called circulating lncRNAs in OS remain elusive. We herein systematically searched, summarized, and discussed all the studies to show the relationship between circulating lncRNA expression levels and OS that can be helpful to address future intervention of circulating lncRNAs in OS management.

The expression levels of thirteen circulating lncRNAs consistently correlated with those measured in OS tissues and have a high potential diagnostic value. In particular, dysregulation of seven lncRNAs (*EPEL*, *FGD5-AS1*, *FAL1*, *HNF1A-AS1*, *LINC01278*, *MALAT1* and *TUG1*) that can be detected in OS blood also coincide with the clinical stage of the disease, metastatic progression or survival, and, above all, with therapeutic response.

Nonetheless, there is still a long way to go to adopt circulating lncRNA in OS clinical practice. In fact, all circulating lncRNAs in OS have only been reported in a single study with the exception of *TUG1*, therefore there is a need for systematic validation studies that investigate multiple lncRNAs with well-characterized and diverse patient samples. In other terms, the research cohort size should be bigger and selection bias should be reduced as much as possible.

This study also highlights a few other critical points that future investigations should consider to support the exploitation of circulating lncRNAs in the management of OS. For instance, blood preparation and endogenous controls in qRT-PCR analysis of circulating lncRNAs still require a standardization methodology. The choice of anticoagulant, the volume required for sample collection, and the temperature for storing the samples need to be more uniform to keep the analysis among different groups consistent. Controversial results might arise through the use of different quantitative standards in qRT-PCR. In this regard, a recent study identified the lncRNA *RP11-204K16.1*, *XLOC_012542*, and *U6* small nuclear RNA as the most stable reference genes for circulating lncRNA analysis in serum for cervical cancer patients [154]. Similarly, a set of these reference genes should be further identified for the quantitative analysis of circulating lncRNAs in OS.

In summary, this study acknowledges both the pros and cons of the use of circulating lncRNAs as biomarkers for OS. The low degree of invasiveness, affordability and time saving procedures make a few circulating lncRNAs, whose expression coincide with the clinical stage of the disease, promising novel biomarkers to add to current clinical practices for the management of OS. However, the lack of standard methodologies and current small sample size still pose a high risk of bias and strongly limit their use.

Several studies have discovered that lncRNAs play critical regulatory roles in the formation of micrometastases through modulating specific signaling pathways in cancer cells [155,156]. Furthermore, the early detection of abnormal expression levels of several serum lncRNAs was linked to the late onset of metastases [157]. As a result, repeated serum lncRNA samples may aid in the detection of micrometastasis, which is only partially detectable using traditional diagnostic approaches. Therefore, along with their application as OS biomarkers, circulating lncRNAs might also be novel candidate targets. In fact, the presence of lncRNAs originating from tumor tissues in the bloodstream strongly suggests a role in cell-to-cell communication that might be relevant to oncogenesis. However, limited studies have been done in this field so far, which mainly point out a role in angiogenesis promotion [158] or in the modulation of how the surrounding cells respond to circulating miRNAs [159].

Overall, the functions of circulating lncRNAs are still unknown. Understanding the mechanisms to regulate the expression levels of circulating lncRNAs might provide new clues on the oncogenesis of OS and new tools in translational medicine

## Figures and Tables

**Table 1:** Characteristic of the included studies.

Study	lncRNA	Sample		Cohort		Methods			Diagnostic Value	Prognostic Value				
		Type	Storage	OS	CTRL	EXCT	MSRM	NRML		Pre-SX	Post-SX	Post-SX + Chemo	Relapse	SRVL
Cai 2017 [73]	*HNF1A-AS1*	serum	n.r	75	24 bone tumour; 21 HC	n.r.	RT-qPCR	n.r.	AUC = 0.845 95% CI 0.759–0.910 *p* value = n.r. sensitivity = 87.2% specificity =88.9%	↑ OS vs. HC *p* < 0.01	↑ OS vs. HC *p* < 0.01	⟷ OS vs. HC ⟷ OS vs. BT *p* value = n.r	↑ vs. Post-S *p* < 0.01	n.r.
Chen 2018 [68]	*EPEL*	serum	LN2	39	42	TRIzol	RT-qPCR	*β-actin*	AUC = 0.8817 95% CI 0.8111–0.9523, *p* < 0.0001 sensitivity = n.r. specificity = n.r.	↑ OS vs. HC *p* < 0.05	n.r.	n.r.	n.r.	↑ expr > ↓ SRVL
Chen 2019 [76]	*HAND2-AS1*	serum	LN2	48	44	n.r.	RT-qPCR	*β-actin*	AUC = 0.8685 95% CI 0.7989–0.9382 *p* < 0.0001 sensitivity = n.r. specificity = n.r.	↓ OS vs. HC *p* < 0.05	n.r.	n.r.	n.r.	n.r.
Han 2017 [77]	*ATB*	serum	−80 °C	60	60	TRIzol	RT-qPCR	*β-actin*	AUC = 0.9236 95% CI 0.8756–0.9716 *p* value = n.r. sensitivity = 83.33% specificity = 90%	↑ OS vs. HC *p* < 0.0001	n.r.	n.r.	n.r.	n.r.
Huo 2017 [78]	*MALAT1*	serum	n.r.	46	40	TRIzol	RT-qPCR	n.r.	AUC = 0.834 95% CI 0.738–0.906 *p*. value = n.r. sensitivity = 80.43% specificity = 72.50%	↑ OS vs. HC *p* < 0.001	n.r.	n.r.	n.r.	↑ expr > ↓ SRVL
Jiang 2020 [70]	*LINC01354*	blood	LN2	30	30	n.r.	RT-qPCR	n.r.	n.r.	↑ OS vs. HC *p* < 0.01	n.r.	n.r.	n.r.	n.r.
Ma 2015 [80]	*TUG1*	plasma	−80 °C	134	36 benign tumour; 40 HC	TRIzol LS	RT-qPCR	*GAPDH*	AUC = 0.849 95% CI = n.r. *p* < 0.001 sensitivity = n.r. specificity = n.r.	↑ OS vs. HC *p* < 0.001)	↑ Pre-S vs Post-S *p* < 0.001	↑ newly diagnosed vs. post-S *p* < 0.001	⟷ newly diagnosed vs. relapse *p* = 0.632)	n.r.
Sheng 2019 [74]	*TUG1*	plasma	n.r.	40	40	TRIzol	RT-qPCR	*β-actin*	AUC = 0.9447 95% CI 0.8943–0.9960 *p* < 0.0001 sensitivity = n.r. specificity = n.r.	↑ OS vs. HC *p* < 0.05	n.r.	n.r.	n.r.	↑ expr > ↓ SRVL
Song 2020 [79]	*FGD5-AS1*	serum	n.r.	97	100	TRIzol	RT-qPCR	*GAPDH*	AUC = 0.893 95% CI = n.r. *p* value = n.r. sensitivity = n.r. specificity = n.r.	↑ OS vs. HC *p* < 0.05	n.r.	n.r.	n.r.	↑ expr > ↓ SRVL
Wang 2017 [72]	*FAL1*	serum	n.r.	42	n.r.	TRIzol	RT-qPCR	*GAPDH*	AUC = 0.839 95% CI 0.772–0.951 *p* value = n.r. sensitivities = 87.2% specificities = 89.1%	↑ OS vs. HC *p* < 0.01	↑ OS vs. HC *p* < 0.01	⟷ OS vs. HC ⟷ OS vs. bBT *p* >0.05	↑ OS vs. Post-S + Chemo *p* < 0.01	n.r.
Wen 2017 [67]	*UCA1*	serum	n.r.	85	74	TRIzol	RT-qPCR	*GAPDH*	AUC = 0.831 95% CI 0.746–0.916 *p* value = n.r. sensitivities = 87.2% specificities = 89.1%	↑ OS vs. HC *p* < 0.01	n.r.	n.r.	n.r.	n.r.
Yang 2019 [75]	*NEF*	plasma	n.r.	49	42	TRIzol	RT-qPCR	*β-actin*	AUC = 0.9176, 95% CI 0.8629–0.9724 *p* value = n.r. sensitivities = n.r. specificities = n.r.	↓ OS vs. HC *p* < 0.05	n.r.	n.r.	n.r.	n.r.
Zhang 2021 [69]	*LINC01278*	serum	fresh	66	66	TRIzol	RT-qPCR	*GAPDH*	AUC = 0.945 95% CI = 0.908–0.982 *p* value <0.001 sensitivity = 90.91% specificity = 88.00%	↑ OS vs. HC *p* < 0.01	n.r.	n.r.	n.r.	↓ expr > ↑ SRVL
Zhao 2019 [71]	*LINK-A*	plasma	n.r.	62	48	RNAzol	RT-qPCR	*GAPDH*	(metastatic cases) AUC = 0.9141, 95% CI 0.8511−0.9771 *p* < 0.0001 sensitivity = n.r. specificity = n.r.	↑ OS vs. HC *p* < 0.05	n.r.	n.r.	n.r.	n.r.

Abbreviations: AUC—area under the curve (from receiver operating characteristic—ROC curve); BT—bone tumor; bBT—benign bone tumor; chemo—chemotherapy; CTRL—control; EXCT—extraction; expr—expression (lncRNA abundance); HC—healthy control; MSRM—measurement; n.r.—not reported; NRML—normalization; OS—osteosarcoma; RT-qPCR—real-time quantitative polymerase chain reaction; SRVL—survival; SX—surgery.

**Table 2:** Characteristics of the retrieved OS-associated circulating lncRNAs.

lncRNA	Gene Name	Chr. Position	Class	Exons	SV	Transcript Length (nt) *	Sub-Cell Localization **	Poly(A)	Orthologs	MOA in OS	Other Disease Annotation ***
*ATB*	*AL589182.3*	14q11.2	ceRNA	3	1	2144	cytoplasm	negative	unk	*ATB* upregulates ZEB1 and ZEB2 expression and promotes OS growth in vivo in an *miR-200s* dependent manner	HCC [95]; NSCLC [96]; CRC [97,98]; ESCC [99]; GBM [100]; RCC [101] GC [102]
*EPEL*	*TENM3-AS1*	4q34.3	antisense	5	2	5306; 3353	nucleus	positive	none	*EPEL* promotes the migration and invasion of OS cells by upregulating ROCK1	GC [103]; LC [87]
*FAL1*	*FALEC*	1q21.2	scaffold	2	1	566	nucleus	unk	none	*FAL1* promotes G2/M transition and regulates EMT, p21, Wnt signaling	OC [104]; CIN [105]; EC [106]; HCC [107]; PC [108]
*FGD5-AS1*	*FGD5-AS1*	3p25.1	antisense	6	5	3805; 3531	nucleus	unk	none	*FGD5-AS1* targets *mir320b* to promote invasion and EMT ability.	NB [109]; ESCC [110]
*HAND2-AS1*	*HAND2-AS1*	4q34.1	antisense	10	11	5156; 743	cytoplasm, exosome	positive	hand2-os1 (*Mm*)	*HAND2-AS1* negatively regulates the expression level of GLUT1 leading to a decline in glucose uptake	EC [111]; CRC [112]; CM [113]; HCC [114]; NSCLC [115]
*HNF1A-AS1*	*HNF1A-AS1*	12q24.31	antisense	1	1	2455	cytoplasm	unk	none	*HNF1A-AS1* negatively regulates *miR-32-5-p* and the Wnt/β-catenin pathway.	HCC [116,117]; ESCC [118] NSCLC [119,120]; UBC [121]; CRC [122,123]; GBM [124]
*LINC01278*	*LINC01278*	Xq11.1	ceRNA	5	6	3006; 831	cytoplasm	unk	none	*LINC01278* suppresses the proliferation and apoptosis of OS cells through mediating *miR-134-5p*/KRAS axis; *LINC01278* sponges the *miR133a-3p* leading to a positive regulation of PTHR1.	HCC [125]; TC [126]
*LINC01354*	*LINC01354*	1q42.2	scaffold	3	1	944	cytoplasm	unk	none	*LINC01354* promote OS cell EMT and invasion through up-regulating integrin β1	CRC [127]
*LINK-A*	*LINC01139*	1q43	ceRNA	2	1	1540	cytoplasm	unk	none	*LINK-A* positively regulates HIF1α to drive metastasis	HCC [128]; OC [129]
*MALAT1*	*LINC00047*	11q13.1	scaffold	2	3	8779; 8,302	nucleus	negative	Malat1 (*Mm*)	*MALAT1* sponges a number of miRNAs to regulate a plethora of cellular targets; scaffold EZH2 to suppress E-cadherin expression	several cancers reviewed in [130,131,132]
*NEF*	*LINC01384*	20p11.21	ceRNA	3	1	675	nucleus	unk	unk	*NEF* reduces the expression of *miRNA-21*	HCC [92]; GC [133]; NSCLC [134]; SCLC [135]; GBM [136]
*TUG1*	*LINC00080*	22q12.2	scaffold, ceRNA	4	8	7653; 5260	cytoplasm, nucleus	positive	tug1 (*Mm*); Tug1 (*Rn*)	*TUG1* positively regulates AKT signaling, *miR-140-5p*/PFN2 axis and RUNX2; *TUG1* positively regulates HIF-1α via silencing of *miR-143-5p*; *TUG1* sponges the *miR153*	several cancers reviewed in [137]
*UCA1*	*LINC00178*	19p13.12	ceRNA	3	1	2314	cytoplasm	positive	none	*UCA1* sponges *miR-513b-5p* leading to an upregulation of E2F5 and Cyclin E; *UCA1* silences the PTEN/AKT signaling pathway	several diseases reviewed in [138,139]

Abbreviations: ceRNA—competing endogenous RNA; CIN—cervical cancer; CM—cardiomyopathies; CRC—colorectal cancer; EC—endometrial cancer; EMT—epithelial-mesenchymal transition; ESCC—esophageal squamous cell carcinoma; GBM—glioma; GC—gastric cancer; HCC—hepatocellular carcinoma; LC—lung cancer; *Mm*—*Mus musculus;* MOA—mode of action; NB—neuroblastoma; NSCLC—non-small cell lung cancer; OC—ovarian cancer; OS—osteosarcoma; PC—prostate cancer; RCC—renal carcinoma; *Rn*—*Rattus norvegicus*; SCLC—small cell lung cancer; SV—splice variants; TC—thyroid carcinoma; UBC—urinary bladder cancer; unk—unknown; * the longest and the shortest SVs are reported. Full review of SV lengths can be found at ensembl.org. ** The subcellular localizations are derived from experimental evidence and/or database annotations, as reported in PubMed, Embase, Scopus and genecards.org. *** Only studies employing human tissues to prove lncRNA causality are shown. Studies reporting a predicted disease-lncRNA association or uncertain causality have been excluded.

## Data Availability

No new data were created in this study. The data presented in this study are openly available in PubMed as listed in the references.

## References

[1] Ottaviani G., Jaffe N. (2009). The etiology of osteosarcoma. Cancer Treat Res..

[2] Gianferante D.M., Mirabello L., Savage S.A. (2017). Germline and somatic genetics of osteosarcoma—Connecting aetiology, biology and therapy. Nat. Rev. Endocrinol..

[3] Lindsey B.A., Markel J.E., Kleinerman E.S. (2017). Osteosarcoma Overview. Rheumatol. Ther..

[4] Klein M.J., Siegal G.P. (2006). Osteosarcoma: Anatomic and histologic variants. Am. J. Clin. Pathol..

[5] Marchandet L., Lallier M., Charrier C., Baud’huin M., Ory B., Lamoureux F. (2021). Mechanisms of Resistance to Conventional Therapies for Osteosarcoma. Cancers.

[6] Gazouli I., Kyriazoglou A., Kotsantis I., Anastasiou M., Pantazopoulos A., Prevezanou M., Chatzidakis I., Kavourakis G., Economopoulou P., Kontogeorgakos V. (2021). Systematic Review of Recurrent Osteosarcoma Systemic Therapy. Cancers.

[7] Misaghi A., Goldin A., Awad M., Kulidjian A.A. (2018). Osteosarcoma: A comprehensive review. SICOT J..

[8] Zhong J., Hu Y., Si L., Geng J., Xing Y., Jiao Q., Zhang H., Yao W. (2020). Clarifying prognostic factors of small cell osteosarcoma: A pooled analysis of 20 cases and the literature. J. Bone Oncol..

[9] Kim S.H., Shin K.H., Moon S.H., Jang J., Kim H.S., Suh J.S., Yang W.I. (2017). Reassessment of alkaline phosphatase as serum tumor marker with high specificity in osteosarcoma. Cancer Med..

[10] Cao L.L., Chen Z., Yue Z., Pei L., Jia M., Wang H., Li T. (2020). Novel classifiers with clinical laboratory parameters for early detection of osteosarcoma. J. Clin. Lab. Anal..

[11] Huang X., Zhang W., Zhang Z., Shi D., Wu F., Zhong B., Shao Z. (2018). Prognostic Value of Programmed Cell Death 1 Ligand-1 (PD-L1) or PD-1 Expression in Patients with Osteosarcoma: A Meta-Analysis. J. Cancer.

[12] Hu T., Yang Q., Xu J., Zhang Z., He N., Du Y. (2015). Role of beta-isomerized C-terminal telopeptides (beta-CTx) and total procollagen type 1 amino-terminal propeptide (tP1NP) as osteosarcoma biomarkers. Int. J. Clin. Exp. Med..

[13] Zuo Z., Hu H., Xu Q., Luo X., Peng D., Zhu K., Zhao Q., Xie Y., Ren J. (2020). BBCancer: An expression atlas of blood-based biomarkers in the early diagnosis of cancers. Nucleic Acids Res..

[14] Szilagyi M., Pos O., Marton E., Buglyo G., Soltesz B., Keseru J., Penyige A., Szemes T., Nagy B. (2020). Circulating Cell-Free Nucleic Acids: Main Characteristics and Clinical Application. Int. J. Mol. Sci..

[15] Dudley J.C., Diehn M. (2021). Detection and Diagnostic Utilization of Cellular and Cell-Free Tumor DNA. Annu. Rev. Pathol..

[16] Alix-Panabieres C., Pantel K. (2021). Liquid Biopsy: From Discovery to Clinical Application. Cancer Discov..

[17] Botti G., Giordano A., Feroce F., De Chiara A.R., Cantile M. (2019). Noncoding RNAs as circulating biomarkers in osteosarcoma patients. J. Cell Physiol..

[18] Aran V., Devalle S., Meohas W., Heringer M., Cunha Caruso A., Pinheiro Aguiar D., Leite Duarte M.E., Moura Neto V. (2021). Osteosarcoma, chondrosarcoma and Ewing sarcoma: Clinical aspects, biomarker discovery and liquid biopsy. Crit. Rev. Oncol. Hematol..

[19] Anfossi S., Babayan A., Pantel K., Calin G.A. (2018). Clinical utility of circulating non-coding RNAs—An update. Nat. Rev. Clin. Oncol..

[20] Guo X., Lv X., Ru Y., Zhou F., Wang N., Xi H., Zhang K., Li J., Chang R., Xie T. (2020). Circulating Exosomal Gastric Cancer-Associated Long Noncoding RNA1 as a Biomarker for Early Detection and Monitoring Progression of Gastric Cancer: A Multiphase Study. JAMA Surg..

[21] Shi J., Li X., Zhang F., Zhang C., Guan Q., Cao X., Zhu W., Zhang X., Cheng Y., Ou K. (2015). Circulating lncRNAs associated with occurrence of colorectal cancer progression. Am. J. Cancer Res..

[22] Abedini P., Fattahi A., Agah S., Talebi A., Beygi A.H., Amini S.M., Mirzaei A., Akbari A. (2019). Expression analysis of circulating plasma long noncoding RNAs in colorectal cancer: The relevance of lncRNAs ATB and CCAT1 as potential clinical hallmarks. J. Cell Physiol..

[23] Benoist G.E., Van Oort I.M., Boerrigter E., Verhaegh G.W., Van Hooij O., Groen L., Smit F., De Mol P., Hamberg P., Dezentje V.O. (2020). Prognostic Value of Novel Liquid Biomarkers in Patients with Metastatic Castration-Resistant Prostate Cancer Treated with Enzalutamide: A Prospective Observational Study. Clin. Chem..

[24] Batista P.J., Chang H.Y. (2013). Long noncoding RNAs: Cellular address codes in development and disease. Cell.

[25] Song Z., Lin J., Li Z., Huang C. (2021). The nuclear functions of long noncoding RNAs come into focus. Noncoding RNA Res..

[26] Uszczynska-Ratajczak B., Lagarde J., Frankish A., Guigo R., Johnson R. (2018). Towards a complete map of the human long non-coding RNA transcriptome. Nat. Rev. Genet..

[27] Quinn J.J., Chang H.Y. (2016). Unique features of long non-coding RNA biogenesis and function. Nat. Rev. Genet..

[28] Jarroux J., Foretek D., Bertrand C., Gabriel M., Szachnowski U., Saci Z., Guo S., Londono-Vallejo A., Pinskaya M., Morillon A. (2021). HOTAIR lncRNA promotes epithelial-mesenchymal transition by redistributing LSD1 at regulatory chromatin regions. EMBO Rep..

[29] Zhao P., Ji M.M., Fang Y., Li X., Yi H.M., Yan Z.X., Cheng S., Xu P.P., Janin A., Wang C.F. (2021). A novel lncRNA TCLlnc1 promotes peripheral T cell lymphoma progression through acting as a modular scaffold of HNRNPD and YBX1 complexes. Cell Death Dis..

[30] Wanowska E., Kubiak M., Makalowska I., Szczesniak M.W. (2021). A chromatin-associated splicing isoform of OIP5-AS1 acts in cis to regulate the OIP5 oncogene. RNA Biol..

[31] Ma P., Pan Y., Yang F., Fang Y., Liu W., Zhao C., Yu T., Xie M., Jing X., Wu X. (2020). KLF5-Modulated lncRNA NEAT1 Contributes to Tumorigenesis by Acting as a Scaffold for BRG1 to Silence GADD45A in Gastric Cancer. Mol. Ther. Nucleic Acids.

[32] Jantrapirom S., Koonrungsesomboon N., Yoshida H., Candeias M.M., Pruksakorn D., Lo Piccolo L. (2021). Long noncoding RNA-dependent methylation of nonhistone proteins. Wiley Interdiscip Rev. RNA.

[33] Hansen T.B., Jensen T.I., Clausen B.H., Bramsen J.B., Finsen B., Damgaard C.K., Kjems J. (2013). Natural RNA circles function as efficient microRNA sponges. Nature.

[34] Munz N., Cascione L., Parmigiani L., Tarantelli C., Rinaldi A., Cmiljanovic N., Cmiljanovic V., Giugno R., Bertoni F., Napoli S. (2021). Exon-Intron Differential Analysis Reveals the Role of Competing Endogenous RNAs in Post-Transcriptional Regulation of Translation. Noncoding RNA.

[35] Zhao Y., Yu Z., Ma R., Zhang Y., Zhao L., Yan Y., Lv X., Zhang L., Su P., Bi J. (2021). lncRNA-Xist/miR-101-3p/KLF6/C/EBPalpha axis promotes TAM polarization to regulate cancer cell proliferation and migration. Mol. Ther. Nucleic Acids.

[36] Li W., Notani D., Ma Q., Tanasa B., Nunez E., Chen A.Y., Merkurjev D., Zhang J., Ohgi K., Song X. (2013). Functional roles of enhancer RNAs for oestrogen-dependent transcriptional activation. Nature.

[37] Huang Z., Yu H., Du G., Han L., Huang X., Wu D., Han X., Xia Y., Wang X., Lu C. (2021). Enhancer RNA lnc-CES1-1 inhibits decidual cell migration by interacting with RNA-binding protein FUS and activating PPARgamma in URPL. Mol. Ther. Nucleic Acids.

[38] Yang M., Lee J.H., Zhang Z., De La Rosa R., Bi M., Tan Y., Liao Y., Hong J., Du B., Wu Y. (2020). Enhancer RNAs Mediate Estrogen-Induced Decommissioning of Selective Enhancers by Recruiting ERalpha and Its Cofactor. Cell Rep..

[39] Lai F., Orom U.A., Cesaroni M., Beringer M., Taatjes D.J., Blobel G.A., Shiekhattar R. (2013). Activating RNAs associate with Mediator to enhance chromatin architecture and transcription. Nature.

[40] Nieminen T., Scott T.A., Lin F.M., Chen Z., Yla-Herttuala S., Morris K.V. (2018). Long Non-Coding RNA Modulation of VEGF-A during Hypoxia. Noncoding RNA.

[41] Zhao H., Liu X., Yu L., Lin S., Zhang C., Xu H., Leng Z., Huang W., Lei J., Li T. (2021). Comprehensive landscape of epigenetic-dysregulated lncRNAs reveals a profound role of enhancers in carcinogenesis in BC subtypes. Mol. Ther. Nucleic Acids.

[42] Vollmers A.C., Covarrubias S., Kuang D., Shulkin A., Iwuagwu J., Katzman S., Song R., Viswanathan K., Vollmers C., Wakeland E. (2021). A conserved long noncoding RNA, GAPLINC, modulates the immune response during endotoxic shock. Proc. Natl. Acad. Sci. USA.

[43] Bian X., Sun Y.M., Wang L.M., Shang Y.L. (2021). ELK1-induced upregulation lncRNA LINC02381 accelerates the osteosarcoma tumorigenesis through targeting CDCA4 via sponging miR-503-5p. Biochem. Biophys. Res. Commun..

[44] Hung T., Wang Y., Lin M.F., Koegel A.K., Kotake Y., Grant G.D., Horlings H.M., Shah N., Umbricht C., Wang P. (2011). Extensive and coordinated transcription of noncoding RNAs within cell-cycle promoters. Nat. Genet..

[45] Zhu G., Luo H., Feng Y., Guryanova O.A., Xu J., Chen S., Lai Q., Sharma A., Xu B., Zhao Z. (2021). HOXBLINC long non-coding RNA activation promotes leukemogenesis in NPM1-mutant acute myeloid leukemia. Nat. Commun..

[46] Tripathi V., Shen Z., Chakraborty A., Giri S., Freier S.M., Wu X., Zhang Y., Gorospe M., Prasanth S.G., Lal A. (2013). Long noncoding RNA MALAT1 controls cell cycle progression by regulating the expression of oncogenic transcription factor B-MYB. PLoS Genet..

[47] Jiang N., Zhang X., Gu X., Li X., Shang L. (2021). Progress in understanding the role of lncRNA in programmed cell death. Cell Death Discov..

[48] Montes M., Lubas M., Arendrup F.S., Mentz B., Rohatgi N., Tumas S., Harder L.M., Skanderup A.J., Andersen J.S., Lund A.H. (2021). The long non-coding RNA MIR31HG regulates the senescence associated secretory phenotype. Nat. Commun..

[49] You Z., Xu S., Pang D. (2020). Long noncoding RNA PVT1 acts as an oncogenic driver in human pan-cancer. J. Cell Physiol..

[50] Han C., Li H., Ma Z., Dong G., Wang Q., Wang S., Fang P., Li X., Chen H., Liu T. (2021). MIR99AHG is a noncoding tumor suppressor gene in lung adenocarcinoma. Cell Death Dis..

[51] Song J.H., Tieu A.H., Cheng Y., Ma K., Akshintala V.S., Simsek C., Prasath V., Shin E.J., Ngamruengphong S., Khashab M.A. (2021). Novel Long Noncoding RNA miR205HG Functions as an Esophageal Tumor-Suppressive Hedgehog Inhibitor. Cancers.

[52] Hong W., Liang L., Gu Y., Qi Z., Qiu H., Yang X., Zeng W., Ma L., Xie J. (2020). Immune-Related lncRNA to Construct Novel Signature and Predict the Immune Landscape of Human Hepatocellular Carcinoma. Mol. Ther. Nucleic Acids.

[53] Du Z., Fei T., Verhaak R.G., Su Z., Zhang Y., Brown M., Chen Y., Liu X.S. (2013). Integrative genomic analyses reveal clinically relevant long noncoding RNAs in human cancer. Nat. Struct. Mol. Biol..

[54] Hua J.T., Chen S., He H.H. (2019). Landscape of Noncoding RNA in Prostate Cancer. Trends Genet..

[55] Chu K.J., Ma Y.S., Jiang X.H., Wu T.M., Wu Z.J., Li Z.Z., Wang J.H., Gao Q.X., Yi B., Shi Y. (2020). Whole-Transcriptome Sequencing Identifies Key Differentially Expressed mRNAs, miRNAs, lncRNAs, and circRNAs Associated with CHOL. Mol. Ther. Nucleic Acids.

[56] Arita T., Ichikawa D., Konishi H., Komatsu S., Shiozaki A., Shoda K., Kawaguchi T., Hirajima S., Nagata H., Kubota T. (2013). Circulating long non-coding RNAs in plasma of patients with gastric cancer. Anticancer Res..

[57] Ren S., Wang F., Shen J., Sun Y., Xu W., Lu J., Wei M., Xu C., Wu C., Zhang Z. (2013). Long non-coding RNA metastasis associated in lung adenocarcinoma transcript 1 derived miniRNA as a novel plasma-based biomarker for diagnosing prostate cancer. Eur. J. Cancer.

[58] Li Q., Shao Y., Zhang X., Zheng T., Miao M., Qin L., Wang B., Ye G., Xiao B., Guo J. (2015). Plasma long noncoding RNA protected by exosomes as a potential stable biomarker for gastric cancer. Tumour Biol..

[59] Dong L., Lin W., Qi P., Xu M.D., Wu X., Ni S., Huang D., Weng W.W., Tan C., Sheng W. (2016). Circulating Long RNAs in Serum Extracellular Vesicles: Their Characterization and Potential Application as Biomarkers for Diagnosis of Colorectal Cancer. Cancer Epidemiol. Biomark. Prev..

[60] Arroyo J.D., Chevillet J.R., Kroh E.M., Ruf I.K., Pritchard C.C., Gibson D.F., Mitchell P.S., Bennett C.F., Pogosova-Agadjanyan E.L., Stirewalt D.L. (2011). Argonaute2 complexes carry a population of circulating microRNAs independent of vesicles in human plasma. Proc. Natl. Acad. Sci. USA.

[61] Redova M., Sana J., Slaby O. (2013). Circulating miRNAs as new blood-based biomarkers for solid cancers. Future Oncol..

[62] Reis E.M., Verjovski-Almeida S. (2012). Perspectives of Long Non-Coding RNAs in Cancer Diagnostics. Front. Genet..

[63] Pritchard C.C., Kroh E., Wood B., Arroyo J.D., Dougherty K.J., Miyaji M.M., Tait J.F., Tewari M. (2012). Blood cell origin of circulating microRNAs: A cautionary note for cancer biomarker studies. Cancer Prev. Res..

[64] Qi P., Zhou X.Y., Du X. (2016). Circulating long non-coding RNAs in cancer: Current status and future perspectives. Mol. Cancer.

[65] Shi T., Gao G., Cao Y. (2016). Long Noncoding RNAs as Novel Biomarkers Have a Promising Future in Cancer Diagnostics. Dis. Markers.

[66] Moher D., Liberati A., Tetzlaff J., Altman D.G., Group P. (2009). Preferred reporting items for systematic reviews and meta-analyses: The PRISMA statement. BMJ.

[67] Wen J.J., Ma Y.D., Yang G.S., Wang G.M. (2017). Analysis of circulating long non-coding RNA UCA1 as potential biomarkers for diagnosis and prognosis of osteosarcoma. Eur. Rev. Med. Pharmacol. Sci..

[68] Chen S., Liu Z., Lu S., Hu B. (2019). EPEL promotes the migration and invasion of osteosarcoma cells by upregulating ROCK1. Oncol. Lett..

[69] Zhang G.F., Zhou B.S., An X.C., An F.M., Li S.H. (2021). LINC01278 is Highly Expressed in Osteosarcoma and Participates in the Development of Tumors by Mediating the miR-134-5p/KRAS Axis. Onco Targets Ther..

[70] Jiang Y., Luo Y. (2020). LINC01354 Promotes Osteosarcoma Cell Invasion by Up-regulating Integrin beta1. Arch. Med. Res..

[71] Zhao B., Liu K., Cai L. (2019). LINK-A lncRNA functions in the metastasis of osteosarcoma by upregulating HIF1alpha. Oncol. Lett..

[72] Wang Y., Zhao Z., Zhang S., Li Z., Li D., Yang S., Zhang H., Zeng X., Liu J. (2018). LncRNA FAL1 is a negative prognostic biomarker and exhibits pro-oncogenic function in osteosarcoma. J. Cell Biochem..

[73] Cai L., Lv J., Zhang Y., Li J., Wang Y., Yang H. (2017). The lncRNA HNF1A-AS1 is a negative prognostic factor and promotes tumorigenesis in osteosarcoma. J. Cell Mol. Med..

[74] Sheng K., Li Y. (2019). LncRNA TUG1 promotes the development of osteosarcoma through RUNX2. Exp. Ther. Med..

[75] Yang Q., Yu H., Yin Q., Hu X., Zhang C. (2019). lncRNA-NEF is downregulated in osteosarcoma and inhibits cancer cell migration and invasion by downregulating miRNA-21. Oncol. Lett..

[76] Chen S., Xu X., Lu S., Hu B. (2019). Long non-coding RNA HAND2-AS1 targets glucose metabolism and inhibits cancer cell proliferation in osteosarcoma. Oncol. Lett..

[77] Han F., Wang C., Wang Y., Zhang L. (2017). Long noncoding RNA ATB promotes osteosarcoma cell proliferation, migration and invasion by suppressing miR-200s. Am. J. Cancer Res..

[78] Huo Y., Li Q., Wang X., Jiao X., Zheng J., Li Z., Pan X. (2017). MALAT1 predicts poor survival in osteosarcoma patients and promotes cell metastasis through associating with EZH2. Oncotarget.

[79] Song Q.H., Guo M.J., Zheng J.S., Zheng X.H., Ye Z.H., Wei P. (2020). Study on Targeting Relationship Between miR-320b and FGD5-AS1 and Its Effect on Biological Function of Osteosarcoma Cells. Cancer Manag. Res..

[80] Ma B., Li M., Zhang L., Huang M., Lei J.B., Fu G.H., Liu C.X., Lai Q.W., Chen Q.Q., Wang Y.L. (2016). Upregulation of long non-coding RNA TUG1 correlates with poor prognosis and disease status in osteosarcoma. Tumour Biol..

[81] Van Heesch S., Van Iterson M., Jacobi J., Boymans S., Essers P.B., De Bruijn E., Hao W., MacInnes A.W., Cuppen E., Simonis M. (2014). Extensive localization of long noncoding RNAs to the cytosol and mono- and polyribosomal complexes. Genome Biol..

[82] Anderson K.M., Anderson D.M., McAnally J.R., Shelton J.M., Bassel-Duby R., Olson E.N. (2016). Transcription of the non-coding RNA upperhand controls Hand2 expression and heart development. Nature.

[83] He Q., Yang S., Gu X., Li M., Wang C., Wei F. (2018). Long noncoding RNA TUG1 facilitates osteogenic differentiation of periodontal ligament stem cells via interacting with Lin28A. Cell Death Dis..

[84] Chen S., Wang J. (2019). HAND2-AS1 inhibits invasion and metastasis of cervical cancer cells via microRNA-330-5p-mediated LDOC1. Cancer Cell Int..

[85] Vickers K.C., Palmisano B.T., Shoucri B.M., Shamburek R.D., Remaley A.T. (2011). MicroRNAs are transported in plasma and delivered to recipient cells by high-density lipoproteins. Nat. Cell Biol..

[86] Bayraktar R., Van Roosbroeck K., Calin G.A. (2017). Cell-to-cell communication: MicroRNAs as hormones. Mol. Oncol..

[87] Park S.M., Choi E.Y., Bae D.H., Sohn H.A., Kim S.Y., Kim Y.J. (2018). The LncRNA EPEL Promotes Lung Cancer Cell Proliferation Through E2F Target Activation. Cell Physiol. Biochem..

[88] Xue M., Chen W., Li X. (2016). Urothelial cancer associated 1: A long noncoding RNA with a crucial role in cancer. J. Cancer Res. Clin. Oncol..

[89] Li Y., Zhang J., Liu Y.D., Zhou X.Y., Chen X., Zhe J., Zhang Q.Y., Zhang X.F., Chen Y.X., Wang Z. (2020). Long non-coding RNA TUG1 and its molecular mechanisms in polycystic ovary syndrome. RNA Biol..

[90] Yang L., Lin C., Liu W., Zhang J., Ohgi K.A., Grinstein J.D., Dorrestein P.C., Rosenfeld M.G. (2011). ncRNA- and Pc2 methylation-dependent gene relocation between nuclear structures mediates gene activation programs. Cell.

[91] Li J., An G., Zhang M., Ma Q. (2016). Long non-coding RNA TUG1 acts as a miR-26a sponge in human glioma cells. Biochem. Biophys. Res. Commun..

[92] Liang W.C., Ren J.L., Wong C.W., Chan S.O., Waye M.M., Fu W.M., Zhang J.F. (2018). LncRNA-NEF antagonized epithelial to mesenchymal transition and cancer metastasis via cis-regulating FOXA2 and inactivating Wnt/beta-catenin signaling. Oncogene.

[93] Song X., Liu Z., Yu Z. (2019). LncRNA NEF is downregulated in triple negative breast cancer and correlated with poor prognosis. Acta Biochim. Biophys. Sin..

[94] Zhou H., Gao Z., Wan F. (2019). Taurine-upregulated gene 1 contributes to cancers through sponging microRNA. Acta Biochim. Biophys. Sin..

[95] Yuan J.H., Yang F., Wang F., Ma J.Z., Guo Y.J., Tao Q.F., Liu F., Pan W., Wang T.T., Zhou C.C. (2014). A long noncoding RNA activated by TGF-beta promotes the invasion-metastasis cascade in hepatocellular carcinoma. Cancer Cell.

[96] Ke L., Xu S.B., Wang J., Jiang X.L., Xu M.Q. (2017). High expression of long non-coding RNA ATB indicates a poor prognosis and regulates cell proliferation and metastasis in non-small cell lung cancer. Clin. Transl. Oncol..

[97] Iguchi T., Uchi R., Nambara S., Saito T., Komatsu H., Hirata H., Ueda M., Sakimura S., Takano Y., Kurashige J. (2015). A long noncoding RNA, lncRNA-ATB, is involved in the progression and prognosis of colorectal cancer. Anticancer Res..

[98] Yue B., Qiu S., Zhao S., Liu C., Zhang D., Yu F., Peng Z., Yan D. (2016). LncRNA-ATB mediated E-cadherin repression promotes the progression of colon cancer and predicts poor prognosis. J. Gastroenterol. Hepatol..

[99] Li Z., Wu X., Gu L., Shen Q., Luo W., Deng C., Zhou Q., Chen X., Li Y., Lim Z. (2017). Long non-coding RNA ATB promotes malignancy of esophageal squamous cell carcinoma by regulating miR-200b/Kindlin-2 axis. Cell Death Dis..

[100] Ma C.C., Xiong Z., Zhu G.N., Wang C., Zong G., Wang H.L., Bian E.B., Zhao B. (2016). Long non-coding RNA ATB promotes glioma malignancy by negatively regulating miR-200a. J. Exp. Clin. Cancer Res..

[101] Xiong J., Liu Y., Jiang L., Zeng Y., Tang W. (2016). High expression of long non-coding RNA lncRNA-ATB is correlated with metastases and promotes cell migration and invasion in renal cell carcinoma. Jpn. J. Clin. Oncol..

[102] Lei K., Liang X., Gao Y., Xu B., Xu Y., Li Y., Tao Y., Shi W., Liu J. (2017). Lnc-ATB contributes to gastric cancer growth through a MiR-141-3p/TGFbeta2 feedback loop. Biochem. Biophys. Res. Commun..

[103] Fu J., Zhao W., Guo D., Li Z. (2020). LncRNA E2F-Mediated Cell Proliferation Enhancing lncRNA Regulates Cancer Cell Behaviors and Affects Prognosis of Gastric Cancer. Dig. Dis. Sci..

[104] Hu X., Feng Y., Zhang D., Zhao S.D., Hu Z., Greshock J., Zhang Y., Yang L., Zhong X., Wang L.P. (2014). A functional genomic approach identifies FAL1 as an oncogenic long noncoding RNA that associates with BMI1 and represses p21 expression in cancer. Cancer Cell.

[105] Naizhaer G., Kuerban A., Meilipa, Kuerban R., Zhou P. (2019). Up-regulation of lncRNA FALEC indicates prognosis and diagnosis values in cervical cancer. Pathol. Res. Pract..

[106] Zheng Q.H., Shi L., Li H.L. (2019). FALEC exerts oncogenic properties to regulate cell proliferation and cell-cycle in endometrial cancer. Biomed. Pharmacother..

[107] Li B., Mao R., Liu C., Zhang W., Tang Y., Guo Z. (2018). LncRNA FAL1 promotes cell proliferation and migration by acting as a CeRNA of miR-1236 in hepatocellular carcinoma cells. Life Sci..

[108] Zhao R., Sun F., Bei X., Wang X., Zhu Y., Jiang C., Zhao F., Han B., Xia S. (2017). Upregulation of the long non-coding RNA FALEC promotes proliferation and migration of prostate cancer cell lines and predicts prognosis of PCa patients. Prostate.

[109] Atmadibrata B., Liu P.Y., Sokolowski N., Zhang L., Wong M., Tee A.E., Marshall G.M., Liu T. (2014). The novel long noncoding RNA linc00467 promotes cell survival but is down-regulated by N-Myc. PLoS ONE.

[110] Gao J., Zhang Z., Su H., Zong L., Li Y. (2020). Long Noncoding RNA FGD5-AS1 Acts as a Competing Endogenous RNA on microRNA-383 to Enhance the Malignant Characteristics of Esophageal Squamous Cell Carcinoma by Increasing SP1 Expression. Cancer Manag. Res..

[111] Yang X., Wang C.C., Lee W.Y.W., Trovik J., Chung T.K.H., Kwong J. (2018). Long non-coding RNA HAND2-AS1 inhibits invasion and metastasis in endometrioid endometrial carcinoma through inactivating neuromedin U. Cancer Lett..

[112] Zhou J., Lin J., Zhang H., Zhu F., Xie R. (2018). LncRNA HAND2-AS1 sponging miR-1275 suppresses colorectal cancer progression by upregulating KLF14. Biochem. Biophys. Res. Commun..

[113] Cheng X., Jiang H. (2019). Long non-coding RNA HAND2-AS1 downregulation predicts poor survival of patients with end-stage dilated cardiomyopathy. J. Int. Med. Res..

[114] Jing G.Y., Zheng X.Z., Ji X.X. (2021). lncRNA HAND2-AS1 overexpression inhibits cancer cell proliferation in hepatocellular carcinoma by downregulating RUNX2 expression. J. Clin. Lab. Anal..

[115] Gao T., Dai X., Jiang Y., He X., Yuan S., Zhao P. (2020). LncRNA HAND2-AS1 inhibits proliferation and promotes apoptosis of non-small cell lung cancer cells by inactivating PI3K/Akt pathway. Biosci. Rep..

[116] Liu Z., Wei X., Zhang A., Li C., Bai J., Dong J. (2016). Long non-coding RNA HNF1A-AS1 functioned as an oncogene and autophagy promoter in hepatocellular carcinoma through sponging hsa-miR-30b-5p. Biochem. Biophys. Res. Commun..

[117] Ding C.H., Yin C., Chen S.J., Wen L.Z., Ding K., Lei S.J., Liu J.P., Wang J., Chen K.X., Jiang H.L. (2018). The HNF1alpha-regulated lncRNA HNF1A-AS1 reverses the malignancy of hepatocellular carcinoma by enhancing the phosphatase activity of SHP-1. Mol. Cancer.

[118] Yang X., Song J.H., Cheng Y., Wu W., Bhagat T., Yu Y., Abraham J.M., Ibrahim S., Ravich W., Roland B.C. (2014). Long non-coding RNA HNF1A-AS1 regulates proliferation and migration in oesophageal adenocarcinoma cells. Gut.

[119] Zhang G., An X., Zhao H., Zhang Q., Zhao H. (2018). Long non-coding RNA HNF1A-AS1 promotes cell proliferation and invasion via regulating miR-17-5p in non-small cell lung cancer. Biomed. Pharmacother..

[120] Wu Y., Liu H., Shi X., Yao Y., Yang W., Song Y. (2015). The long non-coding RNA HNF1A-AS1 regulates proliferation and metastasis in lung adenocarcinoma. Oncotarget.

[121] Feng Z., Wang B. (2018). Long non-coding RNA HNF1A-AS1 promotes cell viability and migration in human bladder cancer. Oncol. Lett..

[122] Zhu W., Zhuang P., Song W., Duan S., Xu Q., Peng M., Zhou J. (2017). Knockdown of lncRNA HNF1A-AS1 inhibits oncogenic phenotypes in colorectal carcinoma. Mol. Med. Rep..

[123] Fang C., Qiu S., Sun F., Li W., Wang Z., Yue B., Wu X., Yan D. (2017). Long non-coding RNA HNF1A-AS1 mediated repression of miR-34a/SIRT1/p53 feedback loop promotes the metastatic progression of colon cancer by functioning as a competing endogenous RNA. Cancer Lett..

[124] Bi Y., Mao Y., Su Z., Du J., Ye L., Xu F. (2021). Long noncoding RNA HNF1A-AS1 regulates proliferation and apoptosis of glioma through activation of the JNK signaling pathway via miR-363-3p/MAP2K4. J. Cell Physiol..

[125] Huang W.J., Tian X.P., Bi S.X., Zhang S.R., He T.S., Song L.Y., Yun J.P., Zhou Z.G., Yu R.M., Li M. (2020). The beta-catenin/TCF-4-LINC01278-miR-1258-Smad2/3 axis promotes hepatocellular carcinoma metastasis. Oncogene.

[126] Lin S., Tan L., Luo D., Peng X., Zhu Y., Li H. (2019). Linc01278 inhibits the development of papillary thyroid carcinoma by regulating miR-376c-3p/DNM3 axis. Cancer Manag. Res..

[127] Li J., He M., Xu W., Huang S. (2019). LINC01354 interacting with hnRNP-D contributes to the proliferation and metastasis in colorectal cancer through activating Wnt/beta-catenin signaling pathway. J. Exp. Clin. Cancer Res..

[128] Li Z.B., Chu H.T., Jia M., Li L. (2020). Long noncoding RNA LINC01139 promotes the progression of hepatocellular carcinoma by upregulating MYBL2 via competitively binding to miR-30 family. Biochem. Biophys. Res. Commun..

[129] Chen Y., Bi F., An Y., Yang Q. (2019). Identification of pathological grade and prognosis-associated lncRNA for ovarian cancer. J. Cell Biochem..

[130] Goyal B., Yadav S.R.M., Awasthee N., Gupta S., Kunnumakkara A.B., Gupta S.C. (2021). Diagnostic, prognostic, and therapeutic significance of long non-coding RNA MALAT1 in cancer. Biochim. Biophys. Acta Rev. Cancer.

[131] Arun G., Aggarwal D., Spector D.L. (2020). MALAT1 Long Non-Coding RNA: Functional Implications. Noncoding RNA.

[132] Sun Y., Ma L. (2019). New Insights into Long Non-Coding RNA MALAT1 in Cancer and Metastasis. Cancers.

[133] Wang X., Jiang X., Zhou L., Wang Z., Huang H., Wang M. (2019). LncRNANEF is involved the regulation of gastric carcinoma cell proliferation by targeting RUNX1. Mol. Med. Rep..

[134] Chang L., Xu W., Zhang Y., Gong F. (2019). Long non-coding RNA-NEF targets glucose transportation to inhibit the proliferation of non-small-cell lung cancer cells. Oncol. Lett..

[135] Wu L., Wang P. (2019). Long non-coding RNA-neighboring enhancer of FOXA2 inhibits the migration and invasion of small cell lung carcinoma cells by downregulating transforming growth factor-beta1. Oncol. Lett..

[136] Huang Q., Chen H., Zuo B., Cheng C., Yu W., Yang Y. (2019). lncRNA NEF inhibits glioma by downregulating TGF-beta1. Exp. Ther. Med..

[137] Ghaforui-Fard S., Vafaee R., Taheri M. (2019). Taurine-upregulated gene 1: A functional long noncoding RNA in tumorigenesis. J. Cell Physiol..

[138] Liu Z., Wang Y., Yuan S., Wen F., Liu J., Zou L., Zhang J. (2021). Regulatory role of long non-coding RNA UCA1 in signaling pathways and its clinical applications. Oncol. Lett..

[139] Hosseini N.F., Manoochehri H., Khoei S.G., Sheykhhasan M. (2021). The Functional Role of Long Non-coding RNA UCA1 in Human Multiple Cancers: A Review Study. Curr. Mol. Med..

[140] Wang J.Y., Yang Y., Ma Y., Wang F., Xue A., Zhu J., Yang H., Chen Q., Chen M., Ye L. (2020). Potential regulatory role of lncRNA-miRNA-mRNA axis in osteosarcoma. Biomed. Pharmacother..

[141] Goodall G.J., Wickramasinghe V.O. (2021). RNA in cancer. Nat. Rev. Cancer.

[142] Ren D., Zheng H., Fei S., Zhao J.L. (2018). MALAT1 induces osteosarcoma progression by targeting miR-206/CDK9 axis. J. Cell Physiol..

[143] Sun Y., Qin B. (2018). Long noncoding RNA MALAT1 regulates HDAC4-mediated proliferation and apoptosis via decoying of miR-140-5p in osteosarcoma cells. Cancer Med..

[144] Zhang J., Piao C.D., Ding J., Li Z.W. (2020). LncRNA MALAT1 facilitates lung metastasis of osteosarcomas through miR-202 sponging. Sci. Rep..

[145] Ye M., Dong S., Hou H., Zhang T., Shen M. (2021). Oncogenic Role of Long Noncoding RNAMALAT1 in Thyroid Cancer Progression through Regulation of the miR-204/IGF2BP2/m6A-MYC Signaling. Mol. Ther. Nucleic Acids.

[146] Fan L., Huang X., Chen J., Zhang K., Gu Y.H., Sun J., Cui S.Y. (2020). Long Noncoding RNA MALAT1 Contributes to Sorafenib Resistance by Targeting miR-140-5p/Aurora-A Signaling in Hepatocellular Carcinoma. Mol. Cancer Ther..

[147] Wang J., Sun G. (2017). FOXO1-MALAT1-miR-26a-5p Feedback Loop Mediates Proliferation and Migration in Osteosarcoma Cells. Oncol. Res..

[148] Zhao H., Hou W., Tao J., Zhao Y., Wan G., Ma C., Xu H. (2016). Upregulation of lncRNA HNF1A-AS1 promotes cell proliferation and metastasis in osteosarcoma through activation of the Wnt/beta-catenin signaling pathway. Am. J. Transl. Res..

[149] He S., Huang Q., Hu J., Li L., Xiao Y., Yu H., Han Z., Wang T., Zhou W., Wei H. (2019). EWS-FLI1-mediated tenascin-C expression promotes tumour progression by targeting MALAT1 through integrin alpha5beta1-mediated YAP activation in Ewing sarcoma. Br. J. Cancer.

[150] Liu K., Huang J., Ni J., Song D., Ding M., Wang J., Huang X., Li W. (2017). MALAT1 promotes osteosarcoma development by regulation of HMGB1 via miR-142-3p and miR-129-5p. Cell Cycle.

[151] Liu J., Li Y., Tong J., Gao J., Guo Q., Zhang L., Wang B., Zhao H., Wang H., Jiang E. (2018). Long non-coding RNA-dependent mechanism to regulate heme biosynthesis and erythrocyte development. Nat. Commun..

[152] Zhang Z., Wu X., Han Q., Huang Z. (2021). Downregulation of long non-coding RNA UCA1 represses tumorigenesis and metastasis of osteosarcoma via miR-513b-5p/E2F5 axis. Anticancer Drugs.

[153] Yang Z., Li X., Yang Y., He Z., Qu X., Zhang Y. (2016). Long noncoding RNAs in the progression, metastasis, and prognosis of osteosarcoma. Cell Death Dis..

[154] Iempridee T., Wiwithaphon S., Piboonprai K., Pratedrat P., Khumkhrong P., Japrung D., Temisak S., Laiwejpithaya S., Chaopotong P., Dharakul T. (2018). Identification of reference genes for circulating long noncoding RNA analysis in serum of cervical cancer patients. FEBS Open Bio.

[155] Wang S., Liang K., Hu Q., Li P., Song J., Yang Y., Yao J., Mangala L.S., Li C., Yang W. (2017). JAK2-binding long noncoding RNA promotes breast cancer brain metastasis. J. Clin. Investig..

[156] Yang F., Huo X.S., Yuan S.X., Zhang L., Zhou W.P., Wang F., Sun S.H. (2013). Repression of the long noncoding RNA-LET by histone deacetylase 3 contributes to hypoxia-mediated metastasis. Mol. Cell.

[157] Zhang L., Niu H., Yang P., Ma J., Yuan B.Y., Zeng Z.C., Xiang Z.L. (2021). Serum lnc34a is a potential prediction biomarker for bone metastasis in hepatocellular carcinoma patients. BMC Cancer.

[158] Dragomir M., Chen B., Calin G.A. (2018). Exosomal lncRNAs as new players in cell-to-cell communication. Transl. Cancer Res..

[159] Ramon Y.C.S., Segura M.F., Hummer S. (2019). Interplay Between ncRNAs and Cellular Communication: A Proposal for Understanding Cell-Specific Signaling Pathways. Front. Genet..

